# Telenursing as an Effective Ally for Improving Patient Outcomes in Diabetes? An Umbrella Review

**DOI:** 10.1002/nop2.70265

**Published:** 2025-07-03

**Authors:** Marika Lo Monaco, Arianna Profeta, Salvatore Corrao

**Affiliations:** ^1^ Department of Health Promotion Sciences, Maternal and Infant Care, Internal Medicine and Medical Specialties [PROMISE] University of Palermo Palermo Italy; ^2^ University Hospital “Paolo Giaccone” Palermo Italy; ^3^ Internal Medicine Unit With Diabetology & Tertiary Foot Care, Rheumatology, Dermatology and Long‐Term Care National Relevance and High Specialization Hospital Trust ARNAS Civico Palermo Italy

**Keywords:** diabetes, nursing, overview of systematic reviews, telehealth, telenursing, umbrella review

## Abstract

**Aim:**

To evaluate telenursing interventions' effectiveness in managing patients with diabetes mellitus.

**Design:**

Overview of systematic reviews.

**Review Methods:**

According to the PRISMA statement, we included studies published from 2005 to 2023 that evaluated telenursing interventions for adult diabetic patients and reported physiological, behavioural, and clinical outcomes. AMSTAR 2 was used to assess the quality of the included studies.

**Data Sources:**

We conducted an umbrella review from July 2023 to May 2024, searching Cochrane Library, PubMed, SCOPUS, and PROSPERO for systematic reviews published from inception to May 10, 2024.

**Results:**

Thirty‐one eligible systematic reviews were identified. Most (*n* = 23) reported positive effects of telenursing interventions (telephone calls, text messaging, mobile software applications, telecoaching) on reducing glycated haemoglobin (HbA1c). Findings on weight loss, hypoglycaemia, and quality of life were heterogeneous. Telenursing interventions promoted self‐management behaviours like medication adherence and dietary improvements. While several studies suggested potential cost‐effectiveness, further studies are needed to explore the long‐term economic impact of telenursing on diabetes management.

**Conclusions:**

Telenursing appears to be a promising approach for improving diabetes management, particularly in self‐management behaviours and HbA1c control. Further research is needed to explore the long‐term sustainability, cost‐effectiveness, and optimal telenursing protocols for diabetes care.

**Implication for the Profession and/or Patient Care:**

This umbrella review highlights the significant role of telenursing in improving diabetes management and patient outcomes. Nurse‐led telehealth interventions have demonstrated their ability to enhance patient self‐management, adherence to treatment plans, and overall well‐being. Additionally, by reducing hospitalisations and healthcare costs and increasing access to care for patients living in rural areas, nurse‐led telehealth interventions represent an effective strategy for improving diabetes care despite initial costs. Given these findings, healthcare providers and policymakers should implement telenursing programs to enhance patient care and system efficiency.

**Reporting Method:**

The authors adhered to the EQUATOR guidelines using the Preferred Reporting Items for Systematic Reviews and Meta‐Analysis (PRISMA) and “the Preferred Reporting Items for Overviews of Reviews Statement” (PRIOR) for the reporting.

**Patient or Public Contribution:**

There was no patient contribution. The European Union—ERDF or ESF, OP Research and Innovation 2014–2020—DM 1062/2021 co‐financed the publication.

**Trial Registration:**

International Prospective Register of Systematic Reviews (PROSPERO) with the protocol number CRD42023427103 https://www.crd.york.ac.uk/PROSPEROFILES/427103_STRATEGY_20230719.pdf

## 1 Introduction

Diabetes mellitus is a heterogeneous disease characterised by hyperglycemia caused by defective insulin secretion or action or both. The global prevalence of diabetes is estimated to affect 537 million adults worldwide, and it represents one of the top ten causes of death. Diabetes significantly impacts patients' lives due to its long‐term complications, and it causes at least 966 billion dollars in health expenditure due to hospital admission for disease exacerbations (Atlas IDF Diabetes 2024).

Nurse‐led patient education interventions on adopting healthy lifestyles, self‐monitoring glycemic values, and the self‐administration of medication, are essential for preventing complications and optimising available healthcare resources (Zhu et al. 2024).

However, access to traditional healthcare services can still be challenging, particularly for patients living in rural areas (Golembiewski et al. 2022).

In recent years, innovative solutions have emerged to fill this gap (Cobry and Wadwa 2022; Glennie et al. 2022). Due to the COVID‐19 pandemic, telehealth has opened up a new path for healthcare professionals to manage non‐communicable diseases, including diabetes, remotely. Telenursing emerged as an essential branch of telehealth. According to this e‐health technology, nursing care is provided via telecommunication tools, such as text message reminders, video consultations, and data transmission (e.g., blood glucose, dietary, drug intake, and patients' lifestyle).

Several systematic reviews have investigated telemedicine‐based management to improve clinical outcomes in diabetic patients. However, some of these reviews focus on a specific type of diabetes (Cobry and Wadwa 2022; Emonena and Ojo 2022), while others do not specifically address the effect of telemedicine delivered solely by nurses on these outcomes (Sotomayor et al. 2023). Furthermore, there are some systematic reviews on the effectiveness of telenursing in diabetes management, but an inclusive and up‐to‐date synthesis of the literature is lacking. This umbrella review aims to fill this gap by providing a rigorous and comprehensive assessment of existing systematic reviews that evaluate the wide range of telenursing interventions (telephone calls, text messages, telemonitoring web‐based platforms, mobile apps, video calls) and their effects on physiological, behavioural and clinical outcomes in patients with Type 1 Diabetes Mellitus (T1DM) and Type 2 Diabetes Mellitus (T2DM). Our results can help policymakers, leaders and other healthcare professionals develop and implement new applications for telenursing in the management of patients with diabetes.

### 1.1 The Review

We conducted an umbrella review to provide an overview of the effectiveness of telenursing interventions in managing diabetes.

Overviews have evolved to respond to the growing need to filter information overload, improve access to targeted information, and inform healthcare decision‐making. Moreover, they can direct the reader to the evidence, summarising existing research or highlighting the absence of evidence (Aromataris et al. 2024).

### 1.2 Aim

To evaluate the effectiveness of telenursing interventions on the management of patients affected by diabetes.

## 2 Methods

### 2.1 Design

This review was conducted under the Preferred Reporting Items for Systematic Reviews and Meta‐Analyses (PRISMA) guideline to ensure consistency and rigour. The Preferred Reporting Items for Overviews of Reviews Statement (PRIOR) was used as a checklist for the reporting. The protocol was previously registered on the International Prospective Register of Systematic Reviews (PROSPERO) with the protocol number CRD42023427103 and is available online.

### 2.2 Analytic Framework

**FIGURE 1:**
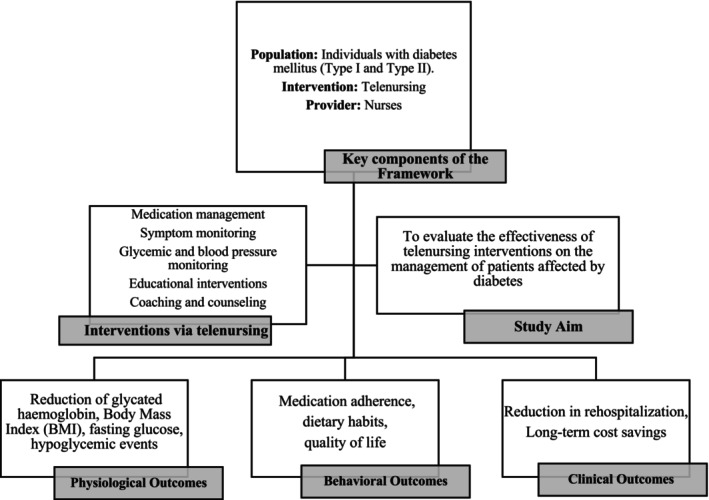
Analytic framework.

We developed an analytical framework (Figure 1) outlining the population, the type of intervention employed, and its providers. The target population consists of individuals with diabetes mellitus. Both type 1 and type 2 diabetes were investigated, as we aimed to make this study as broad and generalisable as possible. First, we established that telenursing was a specific type of telemedicine delivered by nurses through tools such as text messaging, calls, apps, webinars, and more, enabling the achievement of specific health outcomes for patients. We defined telenursing as care provided by a nurse. When this was not explicitly stated, we inferred it by analysing the interventions described in each study. Next, we identified the interventions deliverable through telenursing, including medication management, symptom monitoring, glycaemic and blood pressure monitoring, educational interventions, coaching, and counselling.

The common objective across most studies was to summarise the current components of telenursing and assess their effectiveness. Effectiveness was evaluated based on specific outcomes, categorised into three groups (physiological, behavioural, and clinical outcomes).

### 2.3 Search Methods

From July 2023 to May 2024, searches were performed in various databases: the Cochrane Library, PubMed, SCOPUS, and PROSPERO (Table 1). The search terms were developed by reaching agreement within the research team: “telehealth,” “telenursing,” “diabetes mellitus,” “diabetes,” and “systematic review.”

**TABLE 1:** Database search strategy and retrieved records.

Database	Review period	Search terms	Retrieved records no	Retrieved titles^a^	Excluded studies^a^
Pubmed	2005–2023	(“telehealth”[Title] OR “telenursing”[Title] OR “telenursing”[Title] OR (“telenursing”[MeSH Terms] OR “telenursing”[All Fields]) OR (“telehealth s”[All Fields] OR “telemedicine”[MeSH Terms] OR “telemedicine”[All Fields] OR “telehealth”[All Fields]) OR “telenursing”[All Fields]) AND “systematic review”[Filter] AND “diabet*”[All Fields]	295	Al Ibrahem et al. 2023; Anderson et al. 2022; Baron et al. 2012; Beratarrechea et al. 2014; Bingham et al. 2021; Dawson et al. 2020; Fitzner et al. 2014; Greenwood et al. 2014; Hangaard et al. 2021; Hanlon et al. 2017; Hou et al. 2018; Jaana and Paré 2007; Lee et al. 2017; Lee et al. 2018; Lee et al. 2022; Lewinski et al. 2022; Liang et al. 2011; Marcolino et al. 2013; McDaniel et al. 2022; Paré et al. 2010; Pimouguet et al. 2011; Polisena et al. 2009; Robson and Hosseinzadeh 2021; Rush et al. 2018; Sabahi et al. 2023; Santos et al. 2022; So and Chung 2017; Suksomboon et al. 2014; Udsen et al. 2023; Wu et al. 2010; Yang et al. 2019	Shafiee Hanjani et al. 2020, ^b^; Wade et al. 2010, ^b^; Hazenberg et al. 2019, ^b^; Watzlaf et al. 2017, ^c^; Kruse et al. 2020, ^c^
Cochrane library	2005–2023	telehealth OR telenursing OR telenursing AND diabet*	9		
Scopus	2005–2023	telehealth OR telenursing OR telenursing AND diabet*	54		
Prospero	2005–2023	telehealth OR telenursing OR telenursing AND diabet*	126		
Hand searching	2005–2023	Manual search of reference lists and key journals	9		
Total			493	31	5

^a^ Records included/excluded after reading the full text (Aromataris et al. 2024).

^b^ Economic analysis.

^c^ Not pertinent.

In particular:

Cochrane Library, Scopus, Prospero: telehealth OR telenursing OR telenursing AND diabet*.

Pubmed: (“telehealth”[Title] OR “telenursing”[Title] OR “telenursing”[Title] OR (“telenursing”[MeSH Terms] OR “telenursing”[All Fields]) OR (“telehealth s”[All Fields] OR “telemedicine”[MeSH Terms] OR “telemedicine”[All Fields] OR “telehealth”[All Fields]) OR “telenursing”[All Fields]) AND “systematic review”[Filter] AND “diabet*”[All Fields].

Hand searching was also performed in the included studies' reference lists to ensure the inclusion of potentially relevant studies. After removing the duplicates, one author screened articles by reading the titles and abstracts. Then, two researchers selected reviews for inclusion and agreed on exclusions. Uncertainty was resolved through discussion with comments by a third independent reviewer. Subsequently, one researcher read the full text of the included reviews and extracted data concerning their key characteristics. A second researcher confirmed the inclusion of these reviews and independently extracted data from all the included studies.

### 2.4 Inclusion and/or Exclusion Criteria

We included studies according to the following inclusion criteria: (1) telenursing as an intervention for both T1DM and T2DM; (2) adult population (≥ 18 years); (3) systematic reviews (RS) as a synthesis of all available and relevant evidence which brings together all existing primary studies concerning T1DM and T2DM diabetes. No language restrictions were applied.

We excluded studies whose intervention was used by other professions than nursing (physiotherapists, physicians, psychologists, etc.), studies reporting primary outcomes not consistent with the aim of this overview, non‐full‐text studies that could not be found, and studies concerning the paediatric population.

### 2.5 Search Outcomes

The searched outcomes were: (1) physiological (including reduction of glycated haemoglobin levels, Body Mass Index (BMI), fasting glucose, and hypoglycemic events), (2) behavioural (including, e.g., medication adherence, dietary habits, and quality of life) and (3) clinical (including, e.g., rehospitalisation and costs).

### 2.6 Quality Appraisal

Two authors (PA and LMM) assessed the quality of the included systematic reviews using the Measurement Tool to Checklist Assess Systematic Reviews (AMSTAR 2). AMSTAR 2 is a comprehensive and validated tool for the critical assessment of RS, including both randomised controlled trials and non‐randomised trials to evaluate the effectiveness of healthcare interventions (Table 2). The new version of the tool includes 16 items, such as a priori protocol documentation, scientific quality and selection of study designs to be included, more information on excluded studies, and more detailed consideration of RoB (Risk of Bias). Seven items are considered “critical” and have a greater influence on the evaluation of the overall reliability of the results of each RS; the classification identifies four categories:

- High: 0–1 point of non‐critical weakness;
- moderate: >1 point of non‐critical weakness;
- low: 1 critical point ± non‐critical weaknesses;
- critically low: ≥1 critical point ± non‐critical weaknesses.

**TABLE 2:** Quality assessment of the reviews included according to AMSTAR 2 (a measurement tool to assess systematic reviews).

n.	Authors	Items																Overall rating quality
		1	2*	3	4*	5	6	7*	8	9*	10	11*	12	13*	14	15*	16	
1	Al Ibrahem et al. (2023)	Y	Y	Y	Y	Y	Y	N	PY	PY	N	Y	Y	Y	Y	Y	Y	Low
2	Anderson et al. (2022)	Y	Y	N	PY	Y	Y	N	Y	Y‐	N	Y	Y	Y	Y	Y	Y	Low
3	Baron et al. (2012)	Y	N	Y	PY	Y	Y	N	PY	Y	N	NO MA	NO MA	Y	NO	NO MA	Y	Critically low
4	Beratarrechea et al. (2014)	Y	PY	Y	PY	Y	Y	N	PY	Y	N	NO MA	NO MA	Y	NO	NO MA	Y	Low
5	Bingham et al. (2021)	Y	PY	N	PY	Y	Y	N	N	Y	N	NO MA	NO MA	N	Y	NO MA	Y	Critically low
6	Dawson et al. (2020)	Y	N	N	PY	N	Y	Y	Y	N	Y	NO MA	NO MA	N	N	NO MA	Y	Critically low
7	Fitzner et al. (2014)	Y	N	Y	PY	Y	Y	N	PY	N	N	NO MA	NO MA	N	Y	NO MA	Y	Critically low
8	Greenwood et al. (2014)	Y	N	Y	PY	N	N	N	PY	N	N	NOMA	NOMA	N	N	NO MA	Y	Critically low
9	Hangaard et al. (2021)	Y	Y	PY	PY	Y	Y	N	Y	Y	N	Y	Y	Y	Y	Y	Y	Low
10	Hanlon et al. (2017)	Y	PY	Y	PY	Y	Y	N	Y	PY	N	NO MA	NO MA	Y	Y	NO MA	Y	Low
11	Hou et al. (2018)	Y	Y	Y	PY	Y	Y	N	N	Y	N	Y	Y	Y	Y	N	Y	Critically low
12	Jaana and Paré (2007)	Y	PY	N	Y	Y	Y	N	Y	Y	N	Y	Y	Y	Y	Y	Y	Critically low
13	Lee et al. (2017)	Y	Y	Y	Y	Y	Y	N	Y	Y	N	Y	Y	Y	Y	Y	Y	Low
14	Lee et al. (2018)	Y	PY	N	N	Y	Y	Y	PY	Y	N	Y	Y	Y	Y	Y	Y	Low
15	Lee et al. (2022)	Y	Y	N	PY	Y	Y	N	Y	Y	N	Y	Y	Y	Y	Y	Y	Low
16	Lewinski et al. (2022)	Y	PY	Y	PY	Y	Y	Y	Y	Y	Y	NO MA	NO MA	Y	Y	NO MA	Y	High
17	Liang et al. (2011)	Y	N	Y	PY	Y	Y	N	Y	Y	N	Y	Y	Y	Y	Y	Y	Critically Low
18	Marcolino et al. (2013)	Y	Y	Y	PY	Y	Y	Y	Y	Y	N	Y	Y	Y	Y	Y	Y	Moderate
19	McDaniel et al. (2022)	Y	PY	Y	N	Y	Y	N	PY	Y	N	NO MA	NO MA	Y	Y	NO MA	Y	Low
20	Paré et al. (2010)	Y	PY	Y	Y	Y	Y	Y	PY	Y	N	NO MA	NO MA	Y	Y	NO MA	Y	Moderate
21	Pimouguet et al. (2011)	Y	N	Y	Y	Y	Y	N	Y	Y	N	Y	Y	Y	Y	Y	N	Critically low
22	Polisena et al. (2009)	Y	Y	Y	PY	Y	Y	N	Y	PY	Y	Y	Y	Y	Y	N	Y	Critically low
23	Robson and Hosseinzadeh (2021)	Y	PY	N	N	Y	Y	N	Y	Y	N	Y	Y	Y	Y	N	Y	Critically low
24	Rush et al. (2018)	Y	PY	N	N	Y	Y	N	PY	Y	N	NOMA	NO MA	N	N	NOMA	Y	Critically low
25	Sabahi et al. (2023)	Y	PY	Y	PY	Y	Y	N	PY	PY	N	NOMA	NO MA	Y	Y	NOMA	Y	Low
26	Santos et al. (2022)	Y	Y	N	PY	Y	Y	N	PY	N	N	Y	N	Y	Y	Y	Y	Low
27	So and Chung (2017)	Y	Y	Y	N	Y	Y	N	PY	Y	N	Y	Y	Y	Y	N	Y	Critically low
28	Suksomboon et al. (2014)	Y	Y	Y	Y	Y	Y	N	Y	Y	N	Y	Y	Y	Y	Y	Y	Low
29	Udsen et al. (2023)	Y	Y	Y	Y	Y	Y	Y	PY	Y	N	PY	Y	Y	Y	Y	Y	Moderate
30	Wu et al. (2010)	Y	N	Y	PY	Y	Y	N	Y	Y	N	Y	Y	Y	Y	Y	Y	Low
31	Yang et al. (2019)	Y	Y	Y	N	Y	Y	N	Y	Y	N	Y	Y	Y	Y	Y	Y	Critically low

Abbreviations: N, no; NO MA, no meta‐analysis; PY, partially yes; Y, yes.

### 2.7 Data Abstraction

Two authors extracted data from systematic reviews and discussed the extraction, and disagreement was resolved via discussion with the third author. The data extracted consisted of the database search, study design, the first author and published year, number of included studies and participants, participants' characteristics, country, interventions, follow‐up, outcomes (physiological, behavioural, clinical), primary results and whether it was a meta‐analysis or not. A third researcher independently verified the entire process.

### 2.8 Synthesis

Due to the heterogeneity of data and outcomes, meta‐analysis was not performed in this overview but narratively summarised.

## 3 Results

### 3.1 Search Results

**FIGURE 2:**
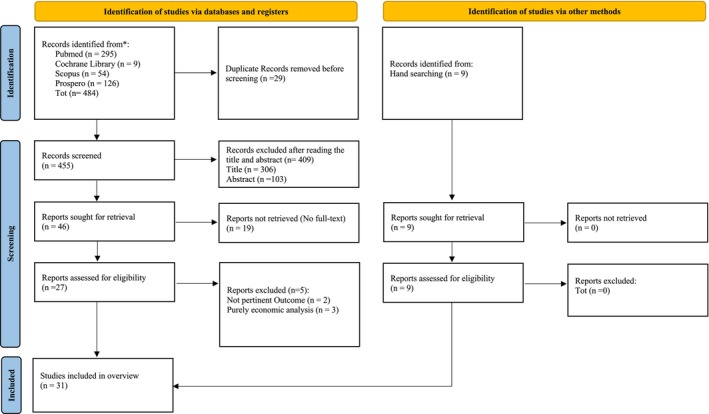
PRISMA flow diagram for the identified systematic reviews.

All records obtained from the initial search were imported to Zotero software to ease the elimination of duplicate studies. After duplicate removal, 455 records potentially meeting the inclusion criteria were identified. As shown in Figure 2, a PRISMA flow chart was used to guide the reporting of the selection process. The screening of the selected titles and abstracts excluded 409 articles. 46 research syntheses were included in the analysis. 19 articles were excluded because no full texts were available, and five were excluded because they were irrelevant. 9 hand searched records were included.

Finally, 31 eligible reviews were identified for methodological quality assessment and narratively summarised (Tables 1 and 3).

**TABLE 3:** Characteristics of the included systematic reviews.

STUDY	Study design	N* participants	Country	Type of population	Framework or theory/concept	Intervention	Follow‐up (duration)	Outcomes: Physiological (P), Behavioural (B), Clinical (C)	RESULTS
Al Ibrahem et al. 2023	RCT	TG: 1096	Italy, Poland, France, Sweden and Spain	T1DM, T2DM and GDM	Not specified in the study	telehome monitoring, web‐based support	6–12 months	HBA1C, FBS, PPBG	HBA1C: The statistical examination displayed no significant heterogeneity (χ^2^ = 1.13, df = 4, *p* = 0.89), with an *I* ^2^ statistic of 0%, indicating uniformity amongst the studies. The overall effect was deemed insignificant (*Z* = 1.39, *p* = 0.17). Indicating that there was no substantial difference in HbA1c levels between the control and intervention groups. FBS: The analysis revealed minimal heterogeneity (Tau^2^ = 1.63; Chi^2^ = 1.01, df = 1, *p* = 0.32) and a significant overall effect (*Z* = 2.43, *p* = 0.02), indicating the superiority of telemedicine over usual care groups in this context. PPBG: The analysis showed no significant heterogeneity (Tau^2^ = 0.00; Chi^2^ = 0.36, df = 1, *p* = 0.55) and an insignificant overall effect (*Z* = 1.11, *p* = 0.27). In this analysis, no significant differences were observed between telemedicine and usual care groups.
Anderson et al. (2022)	RCT	TG: 4426; IG: 2848; CG: 1578.	Not reported	Diabetes	Andersen Behavioural Model	Teleeducation: Diabetes self‐management (DSME).	2 weeks‐12 months	(P)‐ HbA1c	Reduction of HbA1c. Telehealth versus usual care −0.465% (CI: from −0.648% to −0.282%).
Baron et al. (2012)	13 RCT; 2 COT; 5 CBA.	Recruited: 1840; Completed: 1186	Asia (8), Europe (8) and United States (3)	T1DM and T2DM	Not specified in the study	Phone call and PC on self‐monitoring of blood sugar, management of drug therapy, weight and blood pressure control, physical activity education.	3–12 months	(P)‐ HbA1c	Improvement in HbA1c levels
Beratarrechea et al. (2014)	RCT	TG: 4426; IG: 2848; CG: 1578.	Patients live in low‐ and middle‐income countries: China (1), Malaysia (2), Poland (1), India (1) and Uruguay (1)	Diabetes = 6 (Other diseases = 3)	Not specified in the study	Telemonitoring and teleeducation via SMS and calls	Not reported	(P)‐ HbA1c (B)‐ compliance (C)‐ costs and QoL	(P)‐ No significant variations were observed. B‐ A slight improvement in patient compliance was observed. C‐ SMS was found to be less expensive; QoL improved
Bingham et al. (2021)	2RCT; 3 CBA; 2 Retrospective cohort; 1 Matched pre‐post intervention; 1 Cluster randomised trial; 1 Single‐blind randomised trial; 1 Open‐label trial; 1 Prospective matched; 1 Retrospective matched case control study.	TG: 8980; IG: 2879; CG: 6101.	Not reported	T2DM	Not specified in the study	Telephone outreach, text messaging, mobile software applications, and miscellaneous interventions.	3–12 months	(P)‐ blood pressure and lipid values; (B) = medication adherence	(P)‐ A reduction in blood pressure and lipid values was found. (B)‐ Studies have reported positive outcomes demonstrating improved medication adherence
Dawson et al. (2020)	1 RCT; 1 Pre‐post intervention pilot.	TG: 3995	USA (3) = American Indian, native Alaskan, veterans. Canada (1) and Zealand (2 New Maori)	Diabetes = 2 (Other diseases = 4)	Not specified in the study	Intervention technologies ranged from telephone, Internet‐based contact, SMS messaging and video conferencing with a human interaction (nurses).	3–20 months	(P)‐ Hba1c; (B)‐ waist circumference, body weight	(P)‐ Reduction of HbA1c. (B)‐Reduction in body weight, waist circumference
Fitzner et al. (2014)	Meta‐analyses, Systematic Reviews and literature reviews	Not reported.	Not reported	T1DM and T2DM	Not specified in the study	Patient self‐management education and training (DSME/T)	Not reported.	(P)‐ HbA1c; (B)‐ self‐care; (C)‐ costs	(P)‐ Telemedicine that complements usual care is associated with a statistically significant and clinically important decrease in HbA1c. (B)‐Improvements in self‐care behaviours were observed; (C)‐ Telemedicine is cost‐effective. Despite the initial impact, there would be huge economic savings in the long run. The results are very equivocal regarding hospitalisation rates, average hospital days and emergency room visits. Reduction of travel costs incurred to go to specialist facilities.
Greenwood et al. (2014)	RCT	TG: 3744	USA (9), Korea (3), Italy (1), Poland (1) and Spain (1)	T2DM	Not specified in the study	Structured self‐monitoring of blood sugar (SMBG).	3–60 months	(P)‐ HbA1c	(P)‐ Significant decreases in A1C were observed amongst intervention groups compared to control groups.
Hangaard et al. (2021)	RCT	Sample sizes ranged from 17 to 4078, with an average of 251 participants per study.	North America (88), Asia (84), Europe (44), Australia/New Zealand (16), Africa (5) and South America (5)	T2DM	Not specified in the study	Telecoaching, telemonitoring, teleconsultation, telementoring.	1–96 months	(P)‐ HbA1c	Reduction of HbA1c. Telehealth versus usual care: MD of − 0.415% (95% CI = −0.482% to −0.348%)
Hanlon et al. (2017)	Sistematic reviews	Not reported.	North and South America, Europe, Asia and Oceania	T1DM and T2DM	Not specified in the study	Telemonitoring and glicemic control by SMS and follow‐up but telephone calls	Not reported	(P). Hba1c	The highest‐weighted reviews showed that blood glucose telemonitoring with feedback and some educational and lifestyle interventions improved glycemic control in type 2, but not type 1, diabetes
Hou et al. (2018)	RCT	TG: 1550; IG: 907; CG: 643	Finland, Norway, USA (3), Japan, UK (5), Korea, China (2), Canada, Kingdom of Saudi Arabia, Singapore, Poland, India, Italy, France, Australia, Netherland/Not reported.	T1DM and T2DM	Not specified in the study	Use of diabetes apps to support self‐management of the disease.	3–9 months T1DM; 1.5–12 months T2DM	(P)‐ HbA1c	Reduction of HbA1c. Telehealth versus usual care: for T1DM MD −0.49% (95% CI: −0.94 to −0.04). For T2DM MD −0.57% (95% CI: −0.0.82 to −0.32)
Jaana and Paré (2007)	RCT	TG: 1035; IG: 646; CG: 389	North America (10), Europe (6), Asia (1).	Diabetes	Not specified in the study	Telemonitoring of glycemic values	3–15 months	(P)‐ HbA1c and complications; (B)‐ self‐care; (C)‐ costs	(P)‐ Significant decrease in HbA1c and complications associated with diabetes (hypoglycemia and diabetic foot); B‐ Improved self‐management and self‐care. Greater knowledge of one's pathology, change in lifestyle; (C)‐ Reduction of unscheduled annual clinic visits, days of hospitalisation and hospital admission rates. A considerable economic saving for the patient was also estimated
Lee et al. (2022)	RCT	TG: 9677	Not reported	T2DM	Chronic Care Model (Wagner et al.)	Nursing telerehabilitation. Nurse telephone follow‐up and telemonitoring via SMS, website or APP.	Not reported.	P‐ HbA1c; B‐ self‐care; C‐ QoL and costs	(P)‐ No significant effect on HbA1c. Nursing teleeducation vs usual care MD 0,19 (95% CI −0.19 to 0.57, *p* = 32). However, the results are very heterogeneous. (B)‐ Self‐care: Nurse‐led telerehabilitation had a significantly positive effect on improving the self‐care behaviour of patients with diabetes compared to traditional face‐to‐face nursing consultations (Standardised mean difference: 1,20, 95% CI 0.55 to 0.84; *p* < 0.001). QoL: 2 studies reported improvement after receiving telerehabilitation from a nurse. The data on costs and the reduction of improper access to hospitals are very equivocal. An improvement in depression‐related pathology has been demonstrated
Lee et al. (2018)	Systematic reviews	TG: 6509	Not reported.	Diabetes	Not specified in the study	Automatic transmission, in which self‐monitored data is transmitted to a receiving station without interruption via software, the Internet, websites or telephone calls. Patients also received feedback and therapeutic education.	3–60 months	P‐ HbA1c	Small but significant improvement in levels of HbA1c. Telehealth versus usual care MD: −0.55%, 95% CI: −0.73 to −0.36
Lee et al. (2017)	RCT	TG: 20467	North America (54), Europe (16), Asia (34), Australia (2), South America (1)/Not reported	T2DM	Not specified in the study	Variety of platform for communication and delivery of interventions, including telephone, the Internet, SMS. Behavioural therapy, educational counselling, psychosocial support.	6 months or less	(P)‐ HbA1c	Reduction of HbA1c. Telehealth versus usual care −0.43% (95% CI: −0.64% to − 0.21%; *p* < 0.001). There was no evidence that telehealth reduced the risk of hypoglycaemia (risk differences: −0.20%; 95% CI: −0.57% to 0.17%)
Lewinski et al. (2022)	RCT	TG: 466	South Corea, USA (2), Denmark.	T2DM	Telehealth Analytic Framework	Telemonitoring interventions that promoted bidirectional and synchronous communication between patient and healthcare provider via telephone or e‐mail.	< 8 weeks–1 year.	P‐ HbA1c; hospital admissions	(P)‐ There is little certainty that telemedicine has an effect on HbA1c1c level (rated low for serious risk of bias, indirectness and imprecision); (C)‐ There is little certainty that telemedicine has an effect on hospital admissions and has an effect on emergency room attendance.
Liang et al. (2011)	12 RCT; 16 CBA; 12 COT.	TG: 2062; IG: 1025; CG: 1037.	Not reported.	T2DM	Not specified in the study	Mobile phone interventions for self‐management support using SMS and Internet; telemonitoring and continuous education.	3–12 months	P‐ HbA1c	Reduction in glycated haemoglobin in general. Telehealth vs usual care SMD −0.51 (95% CI −0.69 to −0.33), of which reduction of HbA1c for patients with T1DM SMD −0.27 (95% CI −0.54 to −0.01) and reduction of HbA1c for patients with T2DM SMD −0.81 (95% CI −1.11 to −0.50).
Marcolino et al. (2013)	RCT	TG: 4307.	Not reported.	Diabetes	Not specified in the study	Telemonitoring of clinical values, or monitoring combined with education.	6–18 months	P‐ HbA1c; blood pressure; BMI	Telemedicine was associated with a statistically significant and clinically relevant absolute decline in HbA1c level compared to control (mean difference −0.44% [−4.8 mmol/mol] and 95% confidence interval [CI] −0.61 to −0.26% [−6.7 to −2.8 mmol/mol]; *p* < 0.001). LDL‐c was reduced in 6.6 mg/dL (95% CI −8.3 to −4.9; *p* < 0.001), but the clinical relevance of this effect can be questioned. No effects of telemedicine strategies were seen on systolic (−1.6 mmHg and 95% CI −7.2 to 4.1) and diastolic blood pressure (−1.1 mmHg and 95% CI −3.0 to 0.8). The two studies that assessed the effect on BMI demonstrated a tendency of BMI reduction in favour of telemedicine.
McDaniel et al. (2022)	RCT	TG: 6435 TI: 1682 TC: 4753	USA (13), China (3), UK (2); Germany, Netherlands	T1DM and T2DM	Motivational Interviewing (MI)	Combination of face‐to‐face visit and telephone calls or only video calls, web/apps, telephone calls.	3–20 months	(P)‐ HbA1c; (B) – waist circumference, psychological distress, self‐care. (C)‐ QoL, costs	Reduction in glycated haemoglobin, systolic blood pressure (but not diastolic) in patients receiving telehealth interventions. The lipid panel was not affected. B‐ Waist circumference statistically decreased in men. Statistically significant improvements in psychological distress and perceived disease‐related stress were documented. The patient's sense of self‐efficacy with respect to therapy management and physical activity implementation improved. C‐ The QoL is statistically improved. A statistically significant economic impact was found for telemedicine with a lower cost per patient receiving the intervention compared to the control.
Paré et al. (2010)	21 RCT; 3 NON RCT	Not reported.	USA (11), Europe (10), Asia (2), Canada (1)	Diabetes = 24 (Other diseases = 38)	Not specified in the study	Home telemonitoring programmes via structured telephone calls and websites	6–12 months	(P)‐ HbA1c	The results indicated that the intervention, which focused on glycemic control and insulin regulation, was clinically useful in reducing HbA1c.
Pimouguet et al. (2011)	RTC	Sample sizes = 31–1665	USA (26), Canada (5), Europe (3), Asia (7).	T1DM and T2DM	Not specified in the study	Diabetic disease management programmes.	1.5–48 months.	P‐ HbA1c	Disease management programmes resulted in a significant reduction in haemoglobin A1C levels (pooled standardised mean difference between intervention and control groups −0.38 [95% CI −0.47 to −0.29], which corresponds to an absolute mean difference of 0.51%). Hypoglycemic episodes were not systematically assessed.
Polisena et al. (2009)	12 RCT; 9 Observational studies	TG: 5069. Sample size: 19–1665	USA (10), Canada (1), Germany (2), Poland (3), Finland (2), Spain (1), South Korea (3), Italy (1), China (2).	T1DM and T2DM	Not specified in the study	Telemonitoring of glycemic values, symptoms, diet and physical activity levels through messages, video calls or websites	3 months–3 years	(P)‐ HbA1c; (B)‐adherence to treats; (C)‐ hospital admissions	Reduction of glicated haemoglobin (home telemonitoring versus usual care weighted mean difference = −0.22%: 95% CI −0.35 to −0.08). (B)‐ Home telehealth interventions were similar or favourable to UC in terms of QoL, patient satisfaction, adherence to treatment or compliance compared with UC. C‐ The use of health service outcomes indicates that home telehealth interventions help to reduce the number of patients who are hospitalised, number of hospitalisations and BDOC. The number of primary care, specialist, office and home care visits were found to be higher amongst patients using HTM in some studies.
Robson and Hosseinzadeh (2021)	RCT	TG: 9277; IG: 5174; CG: 4103	Canada (1), United Kingdom (4), USA (7), Italy (1), Australia (3), India (2), Denmark (1), Norway (1), Malaysia (2), New Zealand (1), Belgium (1), Canary Islands (1), China (1), Germany (1).	T2DM	Not specified in the study	Telemonitoring, mHealth, telephone communication, virtual (web) consultation and video education. Some studies in used a combination of telemonitoring and mHealth interventions/videoconferencing.	3 months–5 years	(P): HbA1c; (B): Self‐management	Reduction of HbA1c. Telehealth versus usual care: weightesd mean difference: −0.18% (CI −0.35 to −0.01) p = 0.04. Improvements in self‐management‐related behaviours and therapy adherence have been reported in numerous studies. They were evaluated with tools such as Diabetes Self‐Management Questionnaire (DSMQ) and Diabetes Self‐Care Activities Scale.
Rush et al. (2018)	11 RCT; 1 quasi‐experimental; 1 parallel group non‐inferiority study; 2 cohort case control.	TG: 2866	Canada (2), Iran (1), Korea (3), Singapore (1), Taiwan (1), USA (5), Netherlands (3).	Diabetes	Not specified in the study	Teleeducation through 4 delivery modes: web‐based, telephone, video conferencing and secure television.	2–18 months	(P)‐ fasting glucose, 3‐month HbA1c. (B) Knowledge. (C)‐QoL; Hospitalisations	Compared to controls, diabetic intervention groups across all modalities experienced significant improvements in clinical/metabolic indicators with decreases in fasting glucose, 3‐month HbA1c, and postprandial glucose levels. ‐ (B) The level of knowledge of the pathology was comparable between experimental and control groups. (C) No statistical change for QoL. There was no difference in hospitalisations for the three modalities compared to usual care for patients with diabetes.
Sabahi et al. (2023)	RCT	Ig: 1123 CG: 792	(Iran) Tehran, Karaj, Hamedan, Ahvaz and Urmia	T2DM	Not specified in the study	text message service and telephone call to implement patients' self‐management programmes	3 months	Self‐management	Studies in Iran have shown that mHealth has an influential role in controlling the disease and promoting self‐management in type 2 diabetic patients. Many of the interventions and services required for patients with type 2 diabetes can be performed remotely
Santos et al. (2022)	RCT, cross‐sectional, case–control, cohort and clinical.	Not reported.	North America (72), South America (2), Africa (6), Europe (36), Asia (33), Oceania (8), other place (7).	127 T1DM and T2DM (Other diseases: 33)	Not specified in the study	Teleconsultation, teleeducation and telemonitoring	Not reported	P‐ HbA1c; C‐ costs	Reduction of glicated haemoglobin. Telehealth versus usual care weighted mean difference = −0.353% (95% CI −0.45% to −0.25%). Tendency to reduce expenses with health services when Telehealth was used, both for the users and for the health care providers
So and Chung (2017)	RCT	TG: 604	Iran (1), USA (4), South Korea (2)	T1DM and T2DM	Not specified in the study	Automated telephone disease management (ATDM)	12–52 weeks	P‐ HbA1c, FPG, 2HPMG; B: self‐efficacy; C: costs	Reduction of HbA1c statistically significant. Telenursing vs usual care: MD −0.64 (95% CI −1.01 to −0.26). The changes in FPG (fasting plasma glucose) were not very significant: DM −0.26 (95% CI −1.05 to 0.53). Variations in 2HPMG (two‐hour post‐meal glucose) were very significant and in favour of the telenursing group: DM −4.81 (95% CI −6.27 to −3.35). B‐ There was a significant change of knowledge, practice and self‐efficacy levels in the intervention group compared with the control group. C‐ Patients in the intervention group saved travel costs and their time per visit.
Suksomboon et al. (2014)	RCT	TG: 953; IG: 512; CG: 441	USA (3), UK (2)	T1DM and T2DM	Not specified in the study	Telephone contact intervention without electronic transmission of data from patients to healthcare professionals (e.g., Teleeducation, telecoaching)	3–12 months	P‐ HbA1c	Telephone intervention did not significantly improve glycemic control as measured by HbA1c compared to standard clinical care (PMD −0.38%, 95% CI −0.91 to −0.16%). *p* < 0.0001
Udsen et al. 2023	RCT	TG: 1615	Europe, North America, Asia, Australia	T1DM	Not specified in the study	Web portal, telephone, telephone, mail correspondence, smartphone or tablet communication for glycemic, dietary, exercise, medication and fatigue support	3–12 months	P‐ HbA1c, BMI	Treatment effect for HbA1c% favoured telemedicine (mean difference of −0.26% [95% confidence interval: −0.37% to −0.15%]) with moderate effect certainty. Although not significant, secondary outcomes were all in favour of telemedicine except number of severe hypoglycemic events and diabetes knowledge, but the certainty of the evidence for those outcomes was all low or very low.
Wu et al. (2010)	RCT	TG: 1764; IG: 1020; CG: 744	Not reported.	T2DM	Not specified in the study	Structured telephone follow‐up with the aim of educating patients on correct lifestyles, following therapy, controlling blood sugar levels and encouraging self‐management	6–18 months	P‐ HbA1c	The overall standardised effect of telephone follow‐up was equivocal, with endpoint data showing a MD of −0.44 (95% CI –0.93 to 0.06; *p* = 0.08) in favour of the telemedicine intervention
Yang et al. (2019)	11 RCT; 6 quasi‐experimental	TG: 2818; IG: 1410; CG: 1408	Greece, Denmark, Egypt, Belgium, Turkey, USA, UK, Iran, Australia, Thailand and China	Diabetes	Not specified in the study	Automated telephone or nurse‐led follow‐up. SMS service, video conferences, telephone coaching. Communication was synchronous or asynchronous	3–18 months	P‐ HbA1c, BMI, fasting blood sugar; Total cholesterol	The use of telenursing was associated with a significant reduction in HbA1c levels compared to usual care SMD 0.68% (95% CI: 0.33 to 1.03, *p* = 0.0001). BMI had no statistical changes; telenursing vs usual care SMD −0.25% (95% CI: −0.81 to 0.32%, *p* = 0.39). The fasting blood sugar (FBS) had a statistically significant change with a standardised mean difference of −0.19% (95% CI 0.20 to 1.01, *p* = 0.0003). Total cholesterol had no significant changes SMD: −0.09% (95% CI −0.03 to 0.21, *p* = 0.12).

Abbreviations: 2HPMG, two‐hour post‐meal glucose; BMI, Body Mass Index; CBA, controlled before‐after trials; CG, control group; COT, randomised crossover trials; FBS, fasting blood sugar; FPG, fasting plasma glucose; HbA1c, glycated haemoglobin; IG, intervention group; QOL, quality of life; RCT, randomised controlled trials; T1DM, type 1 diabetes mellitus; T2DM, type 2 diabetes mellitus; TG, total group.

### 3.2 Characteristics of the Studies

The 31 reviews included in this overview were published in English from 2005 to 2023 and included adult patients mainly affected by diabetes type I and II. Most studies were conducted in high‐ and middle‐income countries such as the United States, Japan, China, Italy, Germany, France, the United Kingdom and Canada. Others, like India and Africa, were conducted in rural and low‐income areas. The settings of the studies were mainly the patient's home environment, primary care, and residential facilities for the elderly. The common aim of almost all studies was to assess and summarise the effectiveness of telenursing in improving diabetic patient outcomes. Most listed studies do not specify a theoretical or conceptual framework in their publications. However, the study by McDaniel et al. (2022) was based on “Motivational Interviewing” (MI) as a theoretical approach, while Lee et al. (2022) was based on the “Chronic Care Model,” the study of Lewinski et al. (2022) explained its “telehealth analytic framework.” Anderson et al. (2022) applied the “Andersen Behavioural Model” to understand the use of healthcare services.

Evaluated outcomes were physiological (e.g., reduction of glycated haemoglobin levels, Body Mass Index, and hypoglycemic events), behavioural (e.g., medication adherence, dietary habits, and quality of life), and clinical (e.g., rehospitalisation's and costs) (Table 3).

### 3.3 Quality Appraisal

According to AMSTAR 2 criteria, almost all reviews were rated as very low or low quality, except for one of moderate quality and only one considered high quality. Most of these reviews lacked a list of excluded studies and justifications for the exclusion, which was critical flaw no. 7. No studies were excluded after quality appraisal; see Table 2 for details.

### 3.4 Summary of The Included Studies (Table 4)

**TABLE 4:** Telenursing interventions and outcomes: Summary of findings.

Category	Key findings
Interventions & tools	Telenursing involves telemonitoring (clinical values) and telecare (remote nursing services). Tools used: phone calls, text messages, web platforms, mobile apps, video calls. Nurses provided education on self‐management and healthy habits.
Physiological outcomes	Most studies showed telenursing helps reduce HbA1c levels, though one study found no significant association. Some evidence suggested greater effectiveness for type II diabetes. Limited studies on hypoglycemia, weight loss, and BMI reduction. No reported adverse effects.
Behavioural outcomes	Telenursing improved therapy adherence, diet, and self‐management (measured by DSMQ, Diabetes Self‐Care Scale). Significant increase in patient disease awareness. Mixed results on quality‐of‐life improvements.
Clinical & economic impact	Reduced hospital admissions, emergency visits, and length of stay. Some studies showed long‐term cost savings, but start‐up costs remain a factor. Economic benefits require further study.

#### 3.4.1 Interventions, Tools, and Comparison

Telenursing interventions were based on the telemonitoring of clinical values (Greenwood et al. 2014; Hangaard et al. 2021; Marcolino et al. 2013; So and Chung 2017; Hou et al. 2018; Jaana and Paré 2007; Polisena et al. 2009; Wu et al. 2010) or telecare to provide nursing services remotely (Paré et al. 2010; Yang et al. 2019). This latter aspect was important for Indigenous populations, for instance, who have to overcome numerous barriers (including living in remote areas, poverty and lack of financial resources, cultural differences concerning populations, majoritarianism, and lack of trust in the healthcare system all), and contribute to access and utilisation of inadequate health services (Dawson et al. 2020).

The interventions were all conducted and guided by a nurse (Paré et al. 2010; So and Chung 2017; Yang et al. 2019) or by a nurse as the first supplier (Lee et al. 2017).

The analysed telenursing tools mainly concerned telephone calls (Liang et al. 2011; Robson and Hosseinzadeh 2021; So and Chung 2017), text messages, web‐based platforms, mobile apps, and video calls, which were used for communication between patients and healthcare nurses daily, weekly, twice a week, or monthly (Anderson et al. 2022; Hou et al. 2018; McDaniel et al. 2022; Lewinski et al. 2022) or simply as needed.

Nurses were mainly providers of educational interventions about disease‐specific self‐management (Lee et al. 2017, 2022; Rush et al. 2018; Fitzner et al. 2014) or to adopt healthy lifestyle habits (Anderson et al. 2022).

Interventions in some reviews were mainly focused on T2DM (Lee et al. 2017; Hangaard et al. 2021), others on various types of diabetes (Jaana and Paré 2007; Pimouguet et al. 2011; Polisena et al. 2009; McDaniel et al. 2022), while most of the included studies focused on various chronic conditions, including diabetes (Paré et al. 2010; Beratarrechea et al. 2014; Rush et al. 2018; Bingham et al. 2021; Lewinski et al. 2022; Lee et al. 2022).

In all the included studies, interventions were compared by the standard of care, i.e., general assistance.

### 3.5 Type of Outcomes

#### 3.5.1 Physiological Outcomes

Almost all included systematic reviews (Udsen et al. 2023; Anderson et al. 2022; Hanlon et al. 2017; Lee et al. 2018; McDaniel et al. 2022; Robson and Hosseinzadeh 2021; So and Chung 2017; Suksomboon et al. 2014; Hangaard et al. 2021; Baron et al. 2012) evaluated the effectiveness of telenursing interventions in improving glycated haemoglobin (HbA1c) levels.

The HbA1c level is a gold standard in assessing the glycemic status of patients over the past three months. Thus, the reduction in HbA1c levels might reflect telenursing's positive effect on the long‐term care of patients with diabetes (So and Chung 2017).

A systematic review (Suksomboon et al. 2014) found no statistically significant association between telenursing and HbA1c level reduction compared to standard care, particularly for telephone calls without electronic data transmission from patients to healthcare professionals. However, most of the studies mentioned above reported a positive impact of telenursing on HbA1c reduction compared to control in different types of diabetes without specifying whether type 1 or type 2 (Jaana and Paré 2007; Pimouguet et al. 2011; Polisena et al. 2009; McDaniel et al. 2022). One study Liang et al. (2011) suggested that telenursing might be more effective in reducing HbA1c levels for patients with type II diabetes than for patients with type I diabetes. In contrast, Marcolino et al. (2013) reported that telemedicine interventions—including phone calls, text messages, teleconsultation, and telemonitoring—were more effective in reducing glycated haemoglobin in patients with T1DM, with no significant difference between interventions delivered by physicians or nurses.

Wu et al. (2010) proposed that telephone‐based telenursing interventions could be more effective if tailored to individual patient needs and offered greater flexibility.

Only a few studies addressed hypoglycemia risk assessment (Jaana and Paré 2007; Lee et al. 2017). A meta‐analysis by Pimouguet et al. (2011) highlighted that hypoglycemic episodes are often not reported systematically. The limited available data showed no statistically significant difference in hypoglycemia rates between intervention and control groups.

Findings on weight loss and BMI reduction were also heterogeneous. A Chinese SR by Yang et al. (2019) reported no significant effect of telenursing on BMI or total cholesterol level reductions (Wu et al. 2010). None of the reviewed studies reported adverse events related to mortality or morbidity due to telenursing interventions.

#### 3.5.2 BEHAVIOURAL Outcomes

Within behavioural, commonly analysed outcomes in telenursing interventions for diabetes mellitus include adherence to therapy, dietary habits, and improvement in disease self‐management. While HbA1c is the gold standard for long‐term glycemic control, some studies have employed fasting plasma glucose (FPG) to assess short‐term changes in response to interventions, given its sensitivity to recent dietary intake (So and Chung 2017; Robson and Hosseinzadeh 2021; Al Ibrahem et al. 2023). Other studies utilised validated tools like the Diabetes Self‐Management Questionnaire (DSMQ) and the Diabetes Self‐Care Activities Scale (Anderson et al. 2022). Notably, all reviewed studies reported statistically significant improvements in these self‐management indices. Patients receiving telenursing interventions demonstrated a greater awareness of their disease than those receiving traditional care (Lee et al. 2022; Bingham et al. 2021; Sabahi et al. 2023). Some of the included reviews reported a difference in behavioural outcomes for the two types of diabetes. According to Hanlon et al. (2017), blood glucose telemonitoring with feedback and some educational and lifestyle interventions improved glycemic control in type 2, but not type 1, diabetic patients.

Moreover, findings regarding quality of life (QOL) might be clarified. While McDaniel et al. (2022), Lee et al. (2022), and Wong et al. (2022) identified improved quality of life, Lee et al. (2018) reported no significant difference in enhancing the quality of life between groups.

#### 3.5.3 Clinical Outcomes

Several studies supported the potential of telenursing to reduce hospital utilisation and healthcare costs. Polisena et al. (2009) reported that telenursing interventions were associated with fewer hospital admissions due to disease exacerbations, decreased emergency department visits, and shorter hospital stays. Furthermore, five systematic reviews (Beratarrechea et al. 2014; Jaana and Paré 2007; McDaniel et al. 2022; Robson and Hosseinzadeh 2021; So and Chung 2017) explored the economic impact of telenursing on healthcare expenditures. While initial implementation costs can be substantial, studies suggest potential long‐term cost savings. For instance, McDaniel et al. (2022) identified one study demonstrating a statistically significant reduction in cost per patient receiving telenursing compared to controls. Fitzner et al. (2014) also suggested the potential cost‐effectiveness of telenursing but acknowledged that most studies likely underestimate its actual economic benefits due to potential cost reductions realised over time. Consequently, the overall economic benefits of telenursing remain uncertain and require further investigation.

## 4 Discussion

In this study, we conducted an overview of systematic reviews to evaluate the effectiveness of telenursing interventions in diabetes management. The COVID‐19 pandemic demonstrated how important telehealth could be in improving various health outcomes in patients affected by chronic diseases such as diabetes mellitus. It showed its potential for increasing access to healthcare services (Garfan et al. 2021).

Overall, our review showed that the newest nurse‐led approach to diabetes care in monitoring and educating via e‐health technology can be as effective as, or in some cases more effective than, traditional approaches. We found that telenursing positively affects physiological, behavioural, and clinical outcomes. These findings have significant implications for clinical practice and policy, given the rising global prevalence of diabetes and the associated burden on healthcare systems (Atlas IDF Diabetes 2024).

A key physiological outcome found across multiple included reviews on telenursing was the reduction in HbA1c levels, indicating improved long‐term glycemic control. Improving glycemic control has the potential to reduce complications such as cardiovascular disease, neuropathy, and nephropathy in diabetic patients (Sartore et al. 2023). Nonetheless, the effectiveness of telenursing depends on the integration of technological components. Suksomboon et al. (2014) found no significant HbA1c improvement when telephone calls were not supported by electronic data transmission of outcome data, underscoring the importance of real‐time monitoring and feedback. This emphasises the need for healthcare organisations to consider investing in telemonitoring infrastructure—such as secure data exchange platforms—to maximise telenursing interventions' benefits.

Moreover, a physiological impact of telehealth strategies that emerged from this study is hypoglycemia prevention. Our review indicates that continuous glucose monitoring (CGM) systems could be beneficial for detecting fluctuations in blood glucose and preventing severe hypoglycemic events (Murray‐Bachmann et al. 2021; Sotomayor et al. 2023). Despite CGM devices only estimating the future trend of blood sugar and the tendency towards hypoglycemia (Bouillet et al. 2021), integrating CGM into telehealth could reduce hospital admissions related to hypoglycemia (Jaana and Paré 2007; Lee et al. 2017; Pimouguet et al. 2011). Literature on telemonitoring through CGM delivered by nurses is scarce, and future research should focus on defining best practices for incorporating CGM into telenursing protocols, especially for patients at risk of hypoglycemic events.

Another notable advantage of telenursing in the management of diabetes lies in its capacity to enhance patient engagement. Sharing health information with providers fosters patients' sense of responsibility and may motivate them to adhere more closely to their care plans (Glennie et al. 2022). While this effect could be partly attributed to the Hawthorne effect—where individuals alter their behaviour when they know they are being observed—it highlights telenursing's capacity to facilitate ongoing communication, address individual patient needs, and potentially improve self‐management in conditions like diabetes (Caldarone 2021; Robson and Hosseinzadeh 2021). This is particularly relevant for rural individuals who may otherwise struggle with limited access to healthcare services (Golembiewski et al. 2022). In line with this focus on patient involvement, our umbrella review also highlighted how telenursing interventions can promote self‐management behaviours, evidenced by improvements in adherence to therapy, dietary habits, and validated self‐management scores (Rush et al. 2018; Robson and Hosseinzadeh 2021; McDaniel et al. 2022; Sabahi et al. 2023). These enhancements in self‐care are crucial for reducing the long‐term burden of diabetes and align with broader perspectives on telenursing as a tool to empower patients in managing chronic conditions (Creber et al. 2023; Rezende et al. 2023). However, findings on quality of life (QoL) remain heterogeneous. While several studies reported positive changes in chronic patients' QoL (Wong et al. 2022; Ariyanto and Rosa 2024), further research is needed to clarify the factors influencing QoL in telenursing for diabetes management.

Despite encouraging overall results on the effectiveness of telenursing in diabetes management, not all the included studies explicitly compared interventions between different types of diabetes. Some studies (Liang et al. 2011; Hanlon et al. 2017; Yang et al. 2019) reported more pronounced improvements in T2DM populations, possibly due to variations in insulin dependency, disease progression, or patient self‐management needs. On the other hand, Marcolino et al. (2013) observed stronger effects in T1DM, though direct comparisons were limited. Only a few of the included reviews compared the effectiveness of telenursing interventions between T1DM and T2DM. For example, Baron et al. (2012) included both T1DM and T2DM patients but, due to the lack of direct comparisons, concluded that the effectiveness of the intervention on the different types of diabetes could not be determined. These conflicting findings underscore the need for further research to clarify whether interventions should be tailored differently for T1DM and T2DM patients, potentially guided by relevant theoretical models.

While many of the included reviews did not explicitly mention a guiding theoretical framework, some studies explain how nurses apply theory to practice. For instance, Lee et al. (2022) adopted the Chronic Care Model (CCM), which emphasises a proactive approach to care for chronic diseases. In this model, nurses serve as care coordinators to educate patients on self‐management and use telehealth tools for monitoring. McDaniel et al. (2022) utilised Motivational Interviewing (MI) as a theoretical approach to foster behaviour change, with nurses guiding patients in controlling their glycemic levels. Lewinski et al. (2022) introduced a telehealth analytic framework, illustrating how nurse‐led interventions can be tailored to patients. Anderson et al. (2022) employed the Andersen Behavioural Model, examining factors influencing healthcare utilisation, such as perceived need and enabling resources. These examples demonstrate that nurse‐led telehealth strategies are not merely technical solutions but can be firmly rooted in theoretical models that guide intervention design, patient education, and follow‐up. Integrating such frameworks helps nurses translate evidence‐based strategies into consistent clinical practice, enhancing patient outcomes and facilitating standardised evaluation of telehealth programs.

From a policy perspective, our overview found that implementing telenursing strategies for diabetes management could lead to long‐term savings for healthcare systems, primarily through fewer hospital admissions, shorter hospital stays, and reduced travel expenses (Beratarrechea et al. 2014; McLendon 2017; Gentili et al. 2022; Kesavadev and Mohan 2023). While initial costs for technology and staff training may be substantial, these expenses could be offset by improved outcomes and reduced utilisation of in‐person services over time (Jaana and Paré 2007; Fitzner et al. 2014). Although our umbrella review underscores the promise of telenursing for clinical practice and policy, several gaps remain. Further studies are necessary to capture the full spectrum of the cost‐effectiveness of telenursing, to determine its actual value in different types of diabetes management, and to investigate theoretical frameworks on how healthcare professionals can apply theory to practice in telenursing interventions.

## 5 Recommendation for Practice

The literature on telenursing highlights its benefits in improving physiological, behavioural, and clinical outcomes for diabetic patients. Additionally, nurse‐led telehealth interventions can reduce healthcare costs by lowering rehospitalisation rates, emergency room visits, and expenses related to accessing care, particularly for patients in remote areas. Decision‐makers should consider integrating telenursing as a new standard approach in diabetes management.

## 6 Limitations

Several limitations were identified in the reviewed studies. The heterogeneity in the focus on T1DM or T2DM, intervention design, and outcome measures makes it challenging to draw definitive conclusions about the overall effectiveness of telenursing. Additionally, not all studies explicitly compared interventions between T1DM and T2DM, limiting the ability to differentiate their impact on each condition.

The overall quality of the included studies was not high; most were rated critically low or low, and only a few were rated high. Due to the clinical and statistical heterogeneity across the systematic reviews and the varying categories of interventions, we opted for a narrative synthesis rather than a quantitative analysis. This limits the reliability of the evidence regarding the effectiveness of telenursing interventions in diabetes management.

## 7 Conclusion

This umbrella review highlights the effectiveness of telenursing in improving glycemic control, enhancing patient engagement, and potentially reducing healthcare costs for individuals with diabetes. Our research findings suggest that telenursing offers valuable support to traditional treatments, particularly in primary care settings, rural areas, and developing countries. Nurses can translate evidence into practice more effectively by grounding nurse‐led telehealth strategies in robust theoretical frameworks such as the Chronic Care Model or Motivational Interviewing. While these findings have significant policy and clinical implications, further research is needed to establish telenursing interventions' long‐term sustainability and cost‐effectiveness in diabetes management and explore the differential impact on T1DM versus T2DM. Addressing these gaps could enable healthcare systems to harness the full potential of telenursing, ultimately improving the lives of patients with diabetes on a global scale.

## Author Contributions

Corrao Salvatore: Conceptualisation, supervision of all the overview, and final draft review; Marika Lo Monaco and Arianna Profeta (equal): Conceptualisation, database searching, data abstraction, quality appraisal, writing of the original draft, and review and editing with IA (Grammarly) as support.

## Conflicts of Interest

The authors declare no conflicts of interest.

## Supporting information


Data S1.

## Data Availability

Data can be obtained from the corresponding author upon reasonable request.
